# Carrel and His Wonderful Surgery

**Published:** 1913-02

**Authors:** 


### Abstracts and Selections.

### Carrel and His Wonderful Surgery.

Dr. Alexis Carrel is a native of Lyons, France. He is a young man and came to America in order to have better laboratory facilities for experimentation and research. The *Literary Digest* (November 23, 1912) says:

The United States carries off its third Nobel prize this year. To be sure, only one of the winners—our own Theodore—is a native; the others are adopted sons—first, Professor Michelson, and finally Dr. Alexis Carrel, a Frenchman now claiming New York as his home, who has done his work in the Rockefeller Institute in this city. Dr. Carrel has been known for some time through his somewhat sensational experiments in surgical grafting and in maintaining certain parts and organs alive after severance from the bodies where they belonged.

The *London Lancet* says editorially that his discoveries in the surgery of the blood vessels has gone far to revolutionize this branch of medicine and "may almost be said to have created the surgery of the vascular system." It goes on:

Carrel showed that it was possible to make an end-to-end anastomosis of the cut ends of an artery or vein. Many had declared that sutures could not be employed in an artery. In 1903 there was a discussion at a surgical society, in the course of which it was proved that any such attempt to suture vessels was doomed to failure. * * *

When the end-to-end anastomosis of both arteries and veins had been shown to be practicable, several applications were made of it. A lost piece of an artery was replaced by a piece of another vessel, either an artery or a vein, and it was seen that the wall of a vein was quite capable of withstanding the extra pressure of arterial blood. A vein can be anastomosed with an artery, * * * and this operation has met with a certain measure of success. As soon as anastomosis of vessels became certain, the question of the possibility of transplanting organs arose, and Carrel has succeeded in transplanting from one animal to another of the same species several organs, and especially the kidney has been transplanted from one cat to another: the arteries and veins are transplanted at the same time, and are anastomosed to the corresponding vessels, and it has been seen that as soon as the kidneys have been transplanted, and even before the whole of the operation has been concluded, the secretion of urine has begun. * * *

And there is a further advance in the surgery of the blood vessels which is, perhaps, even more surprising. Carrel has shown that a portion of artery may be preserved in cold storage for several days, or even weeks, before transplantation, and yet it will live. Nay, more; although as a rule the tissue of one animal will not grow in the body of an animal of a different species, yet Carrel has found that these portions of blood vessels of dogs can be transplanted from cold storage with success into the bodies of cats. None who have followed with interest these new advances in surgery can doubt that they contain immense possibilities, and the application to man of the methods learned in animals cannot be long delayed. What are the limits of surgery of the vascular system no one can say, but at least it is clear that the Nobel trustees have done well to recognize the work of Alexis Carrel.

The fact that Dr. Carrel's work has been so little known abroad may be explained by the following extract from a notice in *American Medicine* (New York), which dwells on his modesty:

No one * * * can read Dr. Carrel's statements outlining his results without being impressed with the modesty and humility of the man. In this day of extravagant claims and hasty conclusions it is refreshing to find such a spirit as Dr. Carrel has shown in all his undertakings. It is this modesty that has kept so many, even of his colleagues, ignorant of the really marvelous work he was engaged in. Among those who know, however, it is felt that few discoveries since Harvey's concerning the circulation have exerted more far-reaching influences on medicine and surgery than Dr. Carrel's investigations in connection with blood vessel surgery, the transplantation of limbs and organs, and the preservation and growth of tissues outside of and away from the body. As more becomes known of his wonderful skill and daring in experimental surgery and the manipulation of living tissues, the infinite possibilities he has opened up for the future will be readily seen. At the present time Dr. Carrel's results are so startling, and so far removed from the accepted order of things, that it is hard to view them in proper perspective. Indeed, it is not uncommon to hear the opinion expressed that while the things he has done, and is doing, are undeniably wizard-like, they are highly impractical! One surgeon has gone so far as to term such experimental work "acrobatic surgery." Such criticisms and remarks are most unfair, however, and born of a lack of imagination. Many of Dr. Carrel's scientific researches, to be sure, are so unique, so original, and so at variance with established teaching that it is hard to grasp their significance and estimate their true worth. As one studies his humble and modest reports, however, the brilliant character of his attainments begins to appear, and soon it is easy to understand the greatness of his work. No wonder, indeed, that Dr. Carrel's associates are such enthusiastic admirers, for they have had a chance to observe his genius. No one can come in contact with this master workman and note, not only the earnest, whole-souled purpose that dominates the man, but the wonderful technical skill that he is able to command, without acquiring a respect for his character that is only equaled by the admiration his dexterity inspires.

---

When confronted by an irregular rounded growth appearing to spring from the sternomastoid in the middle third of the neck, bear in mind the possibility of a carotid gland tumor.—*American Journal of Surgery.*

---

A small erosion of the trachea may give rise to an hemoptysis, which must be distinguished from a lung hemorrhage by the absence of pulmonary and constitutional symptoms and by the fact that the blood is in small clotted lumps.—*American Journal of Surgery.*

